# Vibration response imaging: a novel noninvasive tool for evaluating the initial therapeutic effect of noninvasive positive pressure ventilation in patients with acute exacerbation of chronic obstructive pulmonary disease

**DOI:** 10.1186/1465-9921-13-65

**Published:** 2012-08-02

**Authors:** Dai Bing, Kang Jian, Sun Long-feng, Tan Wei, Zhao Hong-wen

**Affiliations:** 1 Department of Respiratory Medicine, the First Affiliated Hospital of China Medical University, 155, Nanjing North Street, Heping district, Shenyang 110001, China

**Keywords:** Vibration response imaging, Noninvasive positive pressure ventilation, Initial therapeutic effect, Acute exacerbation of chronic obstructive pulmonary disease

## Abstract

**Background:**

The popular methods for evaluating the initial therapeutic effect (ITE) of noninvasive positive pressure ventilation (NPPV) can only roughly reflect the therapeutic outcome of a patient’s ventilation because they are subjective, invasive and time-delayed. In contrast, vibration response imaging (VRI) can monitor the function of a patient’s ventilation over the NPPV therapy in a non-invasive manner. This study aimed to investigate the value of VRI in evaluating the ITE of NPPV for patients with acute exacerbation of chronic obstructive pulmonary disease (AECOPD).

**Methods:**

Thirty-six AECOPD patients received VRI at three time points: before NPPV treatment (T1), at 15 min of NPPV treatment (T2), and at 15 min after the end of NPPV treatment (T4). Blood gas analysis was also performed at T1 and at 2 hours of NPPV treatment (T3). Thirty-nine healthy volunteers also received VRI at T1 and T2. VRI examination at the time point T2 in either the patients or volunteers did not require any interruption of the on-going NPPV. The clinical indices at each time point were compared between the two groups. Moreover, correlations between the PaCO_2_ changes (T3 vs T1) and abnormal VRI scores (AVRIS) changes (T2 vs T1) were analyzed.

**Results:**

No significant AVRIS differences were found between T1 and T2 in the healthy controls (8.51 ± 3.36 vs. 8.53 ± 3.57, P > 0.05). The AVRIS, dynamic score, MEF score and EVP score showed a significant decrease in AECOPD patients at T2 compared with T1 (P < 0.05), but a significant increase at T4 compared with T2 (P < 0.05). We also found a positive correlation (R^2^ = 0.6399) between the PaCO_2_ changes (T3 vs T1) and AVRIS changes (T2 vs T1).

**Conclusions:**

VRI is a promising noninvasive tool for evaluating the initial therapeutic effects of NPPV in AECOPD patients and predicting the success of NPPV in the early stage.

## Background

Noninvasive positive pressure ventilation (NPPV) has become a standard treatment for acute exacerbation of chronic obstructive pulmonary disease (AECOPD) with a success rate of 80%–85% [1-3]. Proper acknowledgement of its indications is the key to a successful NPPV. However, consensus on indications for NPPV has not been reached so far, and methods for predicting the success rate of NPPV are still lacking [4]. As such, a dynamic decision-making strategy is usually adopted in clinical practice to predict the outcome of NPPV. For instance, NPPV treatment can be tentatively carried out for several hours before a decision can be made on whether it is to be continued or certain NPPV parameters are to be adjusted according to the initial therapeutic effect (ITE). In this regard, ITE is mainly evaluated based on the patient’s clinical picture, such as improvement in dyspnea, vital signs and blood gas analysis [5]. While these clinical observations may reflect the outcome of a patient’s ventilation at hours later, some of them are readily affected by other factors (for example, heart rate and respiratory rate may be affected by fever or emotional stress). Due to its subjective and patient-perceived nature, alleviation of dyspnea may not always be accurate in assessing the ITE. Blood gas analysis is considered more as the “gold standard” but it is time-delayed and invasive. Therefore, these clinical indexes are not optimal indicators or predictors for initial therapeutic effect and ultimately, for the success of NPPV.

Vibration response imaging (VRI) is a non-invasive, non-radioactive bedside imaging technique. VRI enables respiratory sounds to be recorded and quantified objectively, and displayed in two-dimension images with the help of a computer to show the changes in dynamic vibration energy that generates respiratory sounds (lung sounds) in a respiratory cycle. Therefore, VRI can track continuous changes [6-8] of lung sounds during respiratory cycles, and may potentially allow for a quick display of functional changes in a patient on NPPV. Theoretically, evaluation of ITE by using VRI can be more objective and may yield better prediction of a successful NPPV. Little is known so far about the possible role of VRI for these purposes in clinical settings. In this study we compared the VRI indices between healthy volunteers and patients with AECOPD at different NPPV stages, in an effort to explore whether VRI can be used to evaluate the ITE of NPPV in patients with AECOPD and consequently predict the success of NPPV.

## Subjects and methods

### Subjects

This research was done in accordance with the Helsinki Declaration and approved by the Ethical Committee of the First Affiliated Hospital to China Medical University. Informed consents were obtained from all subjects.

#### Volunteers

Inclusion criteria for healthy volunteers were: absence of chronic respiratory disease or cardiovascular disease, negative history of respiratory infection in the previous month, no smoking history, pulmonary function FEV1/FVC >75%, FEV1% >80% and FVC% >80%. Exclusion criteria were: severe deformity of thorax or spinal column, hypertrichosis, skin injury on the back, gestation or lactation, implanted pacemaker or defibrillator. A total of 39 healthy volunteers were selected, with a male to female ratio of 2:1, a mean age of 36.5 ± 13.2 years, a mean height of 1.64 ± 0.08 meters, a mean weight of 67.4 ± 10.9 kg, a mean FEV1% of 98.72 ± 5.34%, and a mean FEV1/FVC of 86.21 ± 6.75%.

#### Patients

Inclusion criteria for AECOPD patients were: (1) a change in baseline dyspnea, cough and/or sputum production exceeding the normal range, showing an acute exacerbation and requiring a change in previously used drugs according to 2007 Global Initiative for Chronic Obstructive Lung Disease (GOLD) guideline [9]; (2) patients diagnosed with type II respiratory failure with PH >7.20; (3) patients who had not received NPPV therapy before; (3) patients who met the basic requirements for NPPV therapy [10], i.e. conscious mind (Kelly-Matthay Score ≤ 3), good compliance, cooperative and comprehensive abilities, little secretion or intact independent cough and expectoration, and relatively stable hemodynamics, and so on. Exclusion criteria were (1) absolute contraindications for noninvasive ventilation [10], including high risk of aspiration and weak airway protective ability, cardiac and respiratory arrest, trauma, burns, physical deformity, or recent surgery on the face or neck or in the oropharyngeal cavity, and obstruction of upper respiratory tract; (2) relative contraindications for noninvasive ventilation [10], including failure to cooperate with NPPV, severe hypoxemia, dysfunction of extrapulmonary visceral organs, intestinal obstruction, and recent operation to the esophagus or epigastrium; (3) patients who refused to receive NPPV therapy; (4) difficulty in V-array sensor placement, such as severe deformity of the thorax or spinal column, polytrichosis, skin lesions on the back, gestation or lactation, implanted pacemaker or defibrillator; (5) failure to receive a VRI examination for at least three times and failure to tolerate NPPV for at least two hours. Finally, 36 patients with AECOPD were recruited from the Respiratory Department and ICU of the Emergency Department, including 19 smokers. Of these patients, the mean smoking index (pack-years) was 24.31 ± 11.52, male to female ratio 2:1, mean age 58.34 ± 14.72 years, mean height 1.62 ± 0.09 meters and mean body-weight 61.5 ± 13.5 kg, respectively. The mean duration of COPD and hospitalization for AECOPD in the previous year was 23.63 ± 13.48 years and 1.93 ± 0.74 times. The mean APACH II score was 16.48 ± 5.24. Lung auscultation identified moist rales in 21 patients and dry rales in 16. The chest X-ray was abnormal in 26 patients (pleural effusion or pulmonary consolidation), including 10 cases of multi-lobular or multi-segmental involvement.

### Experimental procedures

(1) Subjects were recruited according to the inclusion and exclusion criteria after giving their informed consents. (2) They were inquired about their medical and smoking histories and given physical check-up of the chest. (3) Healthy volunteers completed a pulmonary function test and the first VRI examination (T1) while patients with AECOPD completed the first VRI examination and had their vital signs recorded. The comfort level of respiration was assessed by the visual analogue scale (VAS) in the patients [11]; the degree of dyspnea was assessed by the scale for accessory muscle use (SAMU) [12]. Moreover, blood gas analyses were also performed. (4) All subjects received adaptive NPPV treatment for 15 min, and then a second VRI examination was done at 15 min of NPPV treatment (T2). VRI examination at the time point T2 in either the patients or volunteers did not require any interruption of the on-going NPPV. The vital signs, VAS and SAMU scores were recorded in the AECOPD patients. (5) Healthy volunteers completed the experiment while the patients with AECOPD continued the NPPV treatment. The above clinical indices in the AECOPD patients were determined again at 2 hours of NPPV treatment (T3). The blood gas analysis was also performed. (6) NPPV treatment was paused at 2 hours from the start, then a third VRI examination was performed, and the clinical indices were determined at 15 min after the end of NPPV treatment (T4). (7) Based on clinical outcomes, a decision was made on whether the NPPV treatment was to be continued or stopped for an individual AECOPD patient (See Table 1). 

**Table 1:** Experiment flow chart

**Time Points**	**Procedure**	**Healthy volunteers**	**Patients with AECOPD**	**NPPV settings**
Before NPPV Treatment (T1)	Recruitment, informed consent	√	√	S/T Mode, Inspiratory pressure 10 cmH_2_O
	Baseline Assessment (Medical History, Check-up)	√	√	Expiratory pressure 5 cmH_2_O
	Pulmonary function	√		Backup rate 10 times/min
	First VRI examination	√	√	
	Vital signs		√	
	Clinical score		√	
	Blood gas analysis		√	
at 15 min of NPPV treatment (T2)	Vital signs		√	
	Clinical score		√	
	VRI examination	√	√	
at 2 hours of NPPV treatment (T3)	Vital signs		√	Increase the pressure level in the principle of “the highest inspiratory pressure that a patient can endure”
	Clinical score		√	
	Blood gas analysis		√	
at 15 min after the end of NPPV treatment (T4)	Vital signs		√	
	Clinical score		√	
	VRI examination		√	

### Equipment and operation

A noninvasive ventilator (Vision, Philips Respironic, USA) was used for NPPV treatment. The ventilator was set in S/T mode with a 10 cmH_2_O inspiratory pressure, a 5 cmH_2_O expiratory pressure and a 10 cpm backup respiratory rate. The inhaled oxygen concentration in the AECOPD patients was adjusted according to clinical treatment needs (SpO_2_ ≥ 90%). After the AECOPD patients had received initial and adaptive NPPV treatment for 15 min, the pressure level was increased gradually until “the highest inspiratory pressure a patient can endure” was reached after the VRI examination [10]. The VRI_XP_ System (Deep Breeze, Israel, Or-Akiva) was used for VRI examination [13]. The subjects were instructed to breathe naturally by mouth, and their pulmonary sound vibration was recorded during the first 12 s. The signals were analyzed systematically and the data were processed.

### Image analysis

**Figure 1:**
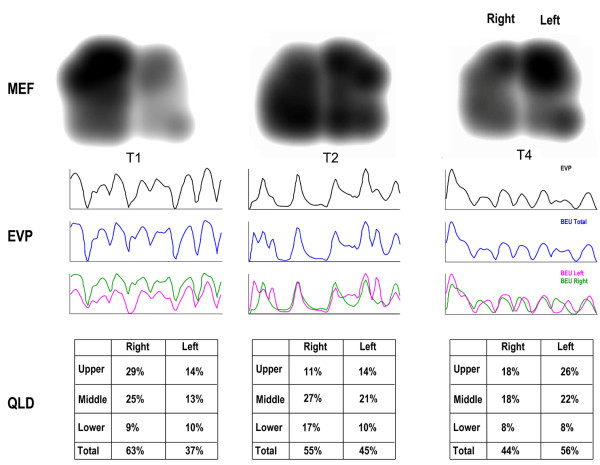
**Typical VRI Image of an AECOPD before, during and stop NPPV treatment.** The typical VRI image changes with NPPV treatment. Panel A (T1), B (T2) and C(T4) represent the MEF (upper row), EVP (middle row) and QLD (lower row) characteristics at the T1,T2 and T4 stages, respectively. The VRI indices during treatment (such as MEF, EVP and QLD) improved dramatically compared with those before treatment; however, after stop NPPV treatment, these indices partly recovered. Abbreviations: T1 = Before NPPV treatment, T2 = at 15 min of NPPV treatment, T4 = at 15 min after the end of NPPV treatment, MEF = maximal energy frame Image, EVP = Expiratory vibration energy peak, QLD = Quantitative lung data.

The output data were presented with grayscale image according to the relative strengths of breath sounds (see Figure 1). Based on different intensities of vibration energy, the representative colors of high data field, low data field and minimum data field are dark color (black), light color (light grey) and white, respectively. The grayscale image, also defined as dynamic vibration energy figure, consists of 71 frames accounting for data in 12 seconds as a whole (each frame contains data in 0.17 seconds). Furthermore, we divided the bilateral lungs into 6 areas, i.e. upper, middle and lower areas on each side. The output of this system contains quantitative lung data (QLD) of these areas in total vibration energy and the vibrational energy curve represented the mean vibration energy that varies with time. The maximal energy frame (MEF) was the frame when the vibration energy reach peak in inspiratory phase. The dry and moist rales could be detected automatically by means of VRI, and marked by red and blue dots in the vibration energy figure correspondingly.

Analysis of the VRI images was performed to check for the following aspects: (1) the vibrational energy curve; (2) Dynamic image (observation from first frame to the last frame one by one); (3) the shape of MEF image; (4) QLD change in different areas; (5) dry and moist rales; (6) Expiratory Vibration energy Peak (EVP), for which we focused our observation on EVP synchronization (ie temporal synchronization of EVP between bilateral lungs) and EVP difference (ie amplitude difference of EVP between bilateral lungs).

### Image scores

Using modified image analysis methods [13-15], three clinicians who had received a systematic training on VRI conducted a blinded image analysis independently. The evaluators had not been informed about the subject’s information, the time points of image acquisition, or the evaluations given by other evaluators. Abnormal VRI image scores (AVRIS) were calculated by adding up the sub-scores as follows (29 points in total): (1) Vibrational energy curve (VEC) score (8 points), including the following abnormal signs (1 point for each sign): unsmooth, inspiratory steep, spike, sag, plateau, single-peak, step (expiratory phase peak value exceeding that of inspiratory phase), and the expiratory phase becoming lower and flat. (2) Dynamic image score (10 points). Image jumping, including a non-vibration type (0 point, the energy center has no quick changing and discontinuous movement), a slight type (1 point, movement of the energy occurs in 1–2 frames occasionally at the inspiratory phase and the expiratory phase), a moderate type (2 points, the movement occurs at the inspiratory phase or the expiratory phase), and a severe type (3 points, the movement occurs at both the inspiratory phase and the expiratory phase). Each of the remaining signs, including dynamic image disorder, desynchronized development, lag, inverse dominance, air trapping, and pneumatocele at the inspiratory phase and the expiratory phase, was scored 1 point. (3) Maximal energy frame (MEF) score (6 points). 0 point is for good MEF image shape, 1 point for average MEF image shape, and 2 points for poor MEF image shape. Each of the abnormal signs, including unsmoothness, midline bending, defect and pneumatocele, was scored 1 point. (4) Rales (2 points). The dry and moist rales were recorded separately, namely, no (0 point) and yes (1 point). (5) Expiratory Vibration energy Peak (EVP). It was scored as per synchronization degree of the left and right curves and amplitude heights (0 point was for synchronization, 1 point for desynchronization at the inspiratory phase or the expiratory phase only, and 2 points for desynchronization at both the inspiratory phase and the expiratory phase. 0 point was for the same amplitude height, and 1 point for the inconformity. The scoring was totally 3 points.)

### Statistics analysis

The software SPSS13.0 was used for statistical analysis. First, inter-evaluator repeatability was tested using intraclass correlation coefficient (ICC). Categorical variables were obtained by Chi-square test or Fisher Exact Probability Test. The VRI scores of the two groups at each time point were tested using two-sample *t*-test, and those between different time points in each group were compared using paired *t*-test or one-way repeated measurements ANOVA. Moreover, Pearson correlation coefficient was used to investigate the relationship between PaCO_2_ changes and AVRIS changes before and after NPPV. A *P* value less than 0.05 was considered statistically significant.

## Results

### The reliability of VRI examination

Thirty-nine healthy volunteers and 36 AECOPD patients successfully underwent a total of 410 VRI examinations without any adverse events. Each examination lasted less than 3 min. VRI records were not influenced by noise from the ward or by the NPPV treatment. ICC showed higher repeatability in the AVRIS between the three evaluators (ICC = 0.935, P < 0.05).

### VRI image characteristics of healthy volunteers

**Figure 2:**
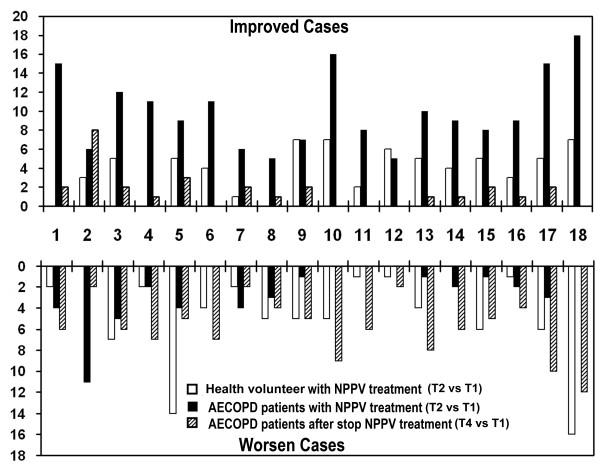
**Individual changes of VRI image score during and at the end of NPPV treatment.** 1 Similarity: The similarity of vibrational energy curve (VEC) among respiratory cycles. 2 Inspiratory steep: Steep peak in VEC caused by sudden increased energy during inspiratory phase. 3 Plateau: Platform in VED, representing little change in vibrational energy. 4 Sag: Concave segment in VED. 5 Low and flat expiration (LFE): low and flat segment in VED during expiration phase. 6 Unsmooth edge of MEF image. 7 Midline bending of MEF image. 8 MEF image defect: abnormal decreased or absent gray-scale intensity in MEF image. 9 Pneumatocele: abnormal increased gray-scale intensity in MEF image. 10 Image jumping: rapid and discontinuous shift of the energy center in dynamic VRI. 11 Occurrence and development disorder: abnormal evolution of the dynamic VRI. In normal subjects, the dynamic VRI appears from upper medial to lower lateral, and disappears from lower lateral to upper medial. 12 Asynchronization: the evolution of bilateral lungs is asynchronous in dynamic VRI. 13 Lag: the dynamic changes of VRI in one lung falls behind another one). 14 Inverse dominance: the dominant side of VRI intensity inverted when breathing cycle changes from inspiratory phase to expiratory phase. 15 Pneumatocele at inspiratory phase: abnormal increased gray-scale intensity in MEF image at inspiratory phase. 16 Pneumatocele at expiratory phase: abnormal increased gray-scale intensity in MEF image at expiratory phase. 17 EVP synchronization: temporal synchronization of EVP between bilateral lungs. 18 EVP difference: amplitude difference of EVP between bilateral lungs.

The curve of vibration energy was smooth and continuous. Imaging of the two lungs was processed simultaneously and the dynamic images were developed synchronously. Bean-shaped MEF displayed smooth, continuous and complete edges and no defect. The sizes and densities of bilateral lungs were roughly equal. The mean total AVRIS was 8.51 ± 3.35 at T1 and 8.53 ± 3.5 at T2, which showed no significant difference between the 2 time points (See Table 2, Figure 2).

**Table 2:** VRI image characteristics in healthy volunteers before and after NPPV treatment

**TP**	**VCG**	**DIG**	**MEFG**	**EVPG**	**RG**	**AVRIS**	**QLDD**
T1	1.67 ± 0.78	4.36 ± 1.78	0.74 ± 0.21	1.15 ± 0.69	0.37 ± 0.15	8.51 ± 3.36	9.74 ± 4.53
T2	2.07 ± 0.98	4.87 ± 1.91	0.76 ± 0.18	1.23 ± 0.58	0.33 ± 0.15	8.53 ± 3.57	9.33 ± 7.65
t	4.55	1.68	0.12	0.36	0.44	0.05	0.71
P	0.04 ^a^	0.20	0.73	0.55	0.52	0.82	0.41

^a^ Significant differences between T1 and T2.

Abbreviation: TP = Time point, T1 = Before NPPV treatment, T2 = 15 min NPPV treatment, VCG = Grading of vibration curve, DIG = Grading of dynamic image, MEFG = Grading of maximal energy frame image, EVPG = Grading of expiratory vibration energy peak, RG = Grading of rales, AVRIS = Abnormal VRI score, QLDD = The QLD changes between the right and left lungs.

### Image characteristics of AECOPD patients

Before NPPV treatment (T1), the VEC was not so smooth, showing step, plateau, sag curve and low and flat expiratory phase curve. The dynamic image was characterized by low imaging synchronization between the left and right lungs, distinct image jumping, numerous dry and moist rales, and unsmooth MEF edges with turgor and defect, with a mean total AVRIS of 18.13 ± 3.67. At 15 min of NPPV treatment (T2), mean total AVRIS (13.16 ± 3.67) was significantly lowered as compared with T1. Sub-scores of AVRIS, such as dynamic score, MEF score and EVP curve score, were significantly lower than those before NPPV treatment (T1) (P < 0.05). The individual change of AVRIS declined in most patients, mainly manifested by plateau, sagging, image jumping and lagging, inverse dominance, synchronicity and equal heights of EVP curves and smoothness of MEF edges. However, the scores of a few patients were increased, as reflected by respiratory creep curve, plateau, low and flat expiratory phase, etc.. At 15 min after the end of NPPV treatment (T4), mean total AVRIS of 15.25 ± 1.26 was increased compared with that at T2. Sub-items of AVRIS such as dynamic score, MEF score, EVP curve score were significantly increased at T4 compared with those at T2 (P < 0.05). the individual change of AVRIS increased in most patients, mainly with manifestation of image jumping, lagging, inverse dominance, synchronicity and equal heights of EVP curves, and smoothness of MEF edge. However, the scores of some items were declined in a few patients, including the respiratory creep curve and the low and flat expiratory phase curve.. The QLD differences between the right and left lungs at T1 (0.34 ± 37.44) were significantly lower than those at T2 (4.11 ± 32.61). The QLD differences at T4 (2.89 ± 35.45) was significantly lower than that at T2 (See Table 3, Figures 1 and 2).

**Table 3:** VRI image characteristics in AECOPD patients before NPPV treatment and after NPPV treatment

**TP**	**VCG**	**DIG**	**MEFG**	**EVPG**	**RG**	**AVRIS**	**QLDD**
T1	3.08 ± 0.92	7.75 ± 1.65	4.21 ± 1.25	2.41 ± 1.12	0.79 ± 0.29	18.13 ± 3.67	0.34 ± 37.44
T2	2.75 ± 1.15	5.83 ± 2.39	2.88 ± 1.59	1.46 ± 0.67	0.83 ± 0.31	13.16 ± 3.67	4.11 ± 32.61
T4	2.78 ± 0.51	6.51 ± 0.58	4.25 ± 0.85	1.75 ± 0.51	0.75 ± 0.31	15.25 ± 1.26	2.89 ± 35.45
F	1.39	5.64	5.76	5.98	0.29	6.18	4.33
P	0.26	<0.01 ^a, b c^	<0.01 ^a,b^	<0.01 ^a,b,c^	0.75	<0.01 ^a,b,c^	0.02 ^a, b^

*Post hoc*: ^a^*P* < 0.05 (T2 vs. T1); ^b^*P* < 0.05 (T4 vs. T2), ^c^*P* < 0.05 (T4 vs.T1).

Abbreviation: TP = Time point, T1 = Before NPPV treatment, T2 = at 15 min of NPPV treatment, T4 = at 15 min after the end of NPPV treatment, VC Score = Score of vibration curve, DI Score = Grading of dynamic image, MEF Score = Score of maximal energy frame image, EVPG = Grading of expiratory vibration energy peak, RG = Grading of rales, AVRIS = Abnormal VRI score, QLDD = The QLD changes between the right and left lungs.

### Clinical improvement in patients with AECOPD

**Figure 3:**
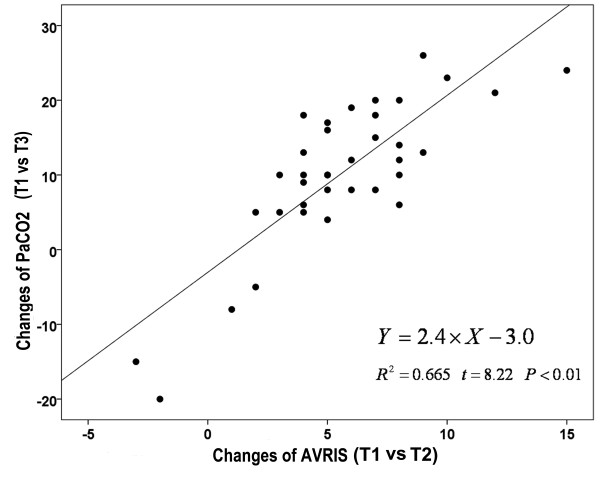
**Correlation between clinical improvement and VRI image score in AECOPD group.** The change of AVRIS (T2 vs T1) is positively correlated with the changes of PaCO_2_ (T3 vs T1).

The heart rate, respiration rate, VAS score and SAMU scores at T2 were significantly lower than those at T1 (P < 0.05), but there were no significant differences between T4 and T2 (P > 0.05). The results of blood gas analysis were significantly improved at 2 hours of NPPV treatment (T3) compared with pretreatment (T1) (P < 0.05). Positive correlation was found between PaCO2 changes (T3 vs T1) and AVRIS changes (T2 vs T1) before and after NPPV treatment (r^2^ = 0.665, P < 0.05) (Figure 3). The NPPV treatment was stopped for four patients whose condition was not improved or even deteriorated as shown by blood gas analysis at two hours after treatment. The total AVRIS changes in the four patients were −4, −2, −1 and 2, respectively. All of them had excessive secretions and poor voluntary cough function. Subsequently, three of them received trachea cannula and one died. NPPV treatment was continued in the remaining 32 patients, none of whom received trachea cannula. They were discharged after their conditions were improved (See Table 4).

**Table 4:** Clinical observation indices of AECOPD patients at different time points

**Group**	**HR (/Min.)**	**MAP (mmHg)**	**RR (/Min.)**	**SPO**_**2**_**(%)**	**VAS**	**AMUS**	**PH**	**PCO**_**2**_**(mmHg)**	**PO**_**2**_**/FiO**_**2**_	**HCO**_**3**_**mmol/l**
T1	109.31 ± 14.62	99.63 ± 10.36	36.35 ± 7.92	94.51 ± 2.52	6.68 ± 0.79	4.21 ± 0.83	7.27 ± 0.09	87.35 ± 9.61	243 ± 32	35 ± 6
T2	98.24 ±7.13	98.45 ± 9.72	25.21 ± 6.43	94.23 ± 2.13	4.31 ± 0.56	3.12 ± 0.48				
T3	97.95 ± 8.52	98.11 ± 10.63	23.24 ± 8.21	94.78 ± 2.41	3.98 ± 0.84	3.03 ± 0.52	7.33 ± 0.12	71.31 ± 7.53	257 ± 24	34 ± 4
T4	99.65 ± 8.72	99.23 ± 11.37	26.54 ± 9.16	94.35 ± 2.31	4.82 ± 0.82	3.64 ± 0.68				
F	4.66	0.01	2.25	0.05	5.35	2.36	0.69	6.36	1.20	0.53
P	0.016 ^a,c^	0.99	0.12 ^a c^	0.95	<0.01 ^a, c^	0.11 ^a, c^	0.41	0.02 ^d^	0.26	0.61

*Post hoc*: ^a^*P* < 0.05 (T2 vs. T1); ^b^*P* < 0.05 (T4 vs. T2), ^c^*P* < 0.05 (T4 vs.T1), ^d^*P* < 0.05 (T3 vs.T1).

Abbreviations: TP = Time point, T1 = Before NPPV treatment, T2 = at 15 min of NPPV treatment, T3 = at 2 hours of NPPV treatment, T4 = at 15 min after the end of NPPV treatment, HR = heart rate, MAP = mean arterial pressure, RR = respiratory rate, SPO_2_ = Pulse oxygen saturation, VAS = visual analogue scale grading, AMUS = scale for accessory muscle use, PH = Potential Of Hydrogen, PCO_2_ = partial pressure of carbon dioxide.

## Discussion

Since a breath sound is highly related to the gas distribution in the lungs, it can be used to determine the ventilation condition at different areas of the lungs [16]. Airway obstruction or stenosis can result in abnormal function of regional ventilation and a corresponding change in breath sounds [17]. With the aid of computer, VRI enables the lung sounds to be objectively recorded, quantified and imaged in two dimensions so that dynamic changes in ventilation can be “visualized”. As an effective ventilation support method, NPPV can improve the ventilatory function of AECOPD patients. Therefore, it is possible for VRI to quickly display the changes of a patient’s ventilatory function over the NPPV treatment.

This hypothesis was confirmed by the present study, in which, the features of VRI in patients with AECOPD were obviously different from those in healthy volunteers. Distinct decline in total AVRIS was found at 15 min of NPPV treatment (T2) compared with T1, and was correlated to the improvement of patients’ clinical indexes (such as PCO_2_). Blood gas analysis (two hours later) is considered more as the “gold standard” for evaluating the initial therapeutic effect. Positive correlation was found between changes in PaCO_2_ (T3 vs T1) and changes in AVRIS (T2 vs T1). Therefore, VRI may be also an effective method for evaluating the ITE. In addition, significant changes can be noted earlier in VRI image (15 minutes later, T2) than with blood gas analysis (two hours later, T3). Although certain clinical indexes at 15 minutes of NPPV (such as HR, RR, VAS score and SAMU scores) were also significantly lowered than baseline (T1), those were more readily affected by other factors and were with a subjective, patient-perceived nature compared with VRI.

NPPV treatment was only a ventilation-supporting approach. When NPPV treatment was paused, the patients’ clinical condition should have resumed what it was before the treatment unless the primary disease was cured. In our study, no obvious change were found in the clinical indexes at T4 vs T2, but there was a distinct difference in abnormal VRI image score (T4 vs T2) among the patients. From another point of view, this suggested that VRI could quickly reflect the effects of NPPV treatment on patients’ ventilatory function and was more sensitive than commonly used clinical indexes. We further found that VRI changes were in accordance with the physiological changes that reflected the ventilatory function during the NPPV treatment.

After NPPV treatment, the VRI images of 14 healthy volunteers showed a low and flat expiration (LFE), namely, the vibration energy of the expiration phase appeared low and flat. It may be caused by a positive end-expiratory pressure (PEEP), which generates a physiological effect similar to intrinsic PEEP during expiration. Therefore, the limited expiratory airflow was displayed in the VEC as a low and flat expiratory phase. Intrinsic PEEP is commonly found in AECOPD patients and is considered as the primary cause of expiration airflow limitation, inspiration load increase and respiration failure [18]. An important mechanism by which NPPV improves ventilatory function is to confront intrinsic PEEP with a proper level of external PEEP [19]. In this research, only a few patients obtained improved LFE in VRI images. The possible reason is that the relative low PEEP (5 cmH_2_O) could not completely counteract the intrinsic PEEP of AECOPD patients at the initial stage of NPPV treatment. As found by Shaul Lev, et al, a VRI examination could monitor the physiological influences on patients with mechanical ventilation by different PEEP levels [20].

MEF can provide more detailed information about respiratory sounds as it reflects air distribution at the peak of air vibration in the lung. Abnormal MEF shapes reflect air distribution of regional airway obstruction or stenosis [21]. The vortex flow and partial dysfunction of ventilation in COPD patients were proved by dynamic magnetic resonance imaging with He-3 absorption, accounting for the unsmooth edge of MEF image in AECOPD patients [22]. Previous researches showed that there existed pulmonary alveoli with different time constants. The filling velocity of “quick” and “slow” pulmonary alveoli varied during normal breathing [23]. The initial high-speed airflow generated by a positive pressure may reach the central respiratory tract rapidly during NPPV treatment. Therefore, it will take more time for the airflow to be uniformly distributed in “quick” and “slow” pulmonary alveoli. In our research, 11 patients had improved MEF images after NPPV treatment, similar to the MEF characteristics in patients with asthma after absorption of bronchodilator [15]. Because structural improvements in the AECOPD lungs cannot be achieved in a short time, the dynamic images of the patients tended to restore to their initial pretreatment conditions once the NPPV treatment was terminated. Dellinger et al found that a VRI examination can estimate the influences of different mechanical ventilation modes on the energy distribution in patients who received the invasive mechanical ventilation [24]. In this study, we also observed the influences of NPPV on the vibration energy distribution in the bilateral lungs. QLD values of the left and right lungs were approximately equal in normal subjects. The QLD values of the left and right lungs in AECOPD patients, however, were found to be about 10% deviated from each other. After NPPV treatment, the QLD values of the left and right lungs were close to those in normal subjects (4%), suggesting that NPPV therapy can improve the air distribution in the lungs, resulting in more uniform air distribution in the bilateral lungs.

The breathing airflow of a normal subject produces continuous vibration when passing through an uninterrupted branching airway. Since AECOPD patients usually suffer inflammation and sputum blocking, their uneven airflow resistance will result in discontinuous vibration energy in both time and space. Such discontinuity is reflected by unsmooth, sagging and step of the VEC, or by image jumping, lagging and inverse dominance in the dynamic vibration energy image [25]. The mechanism by which NPPV improves ventilatory function also involves the positive pressure ventilation which can overcome the airway resistance and increase the alveolar ventilation [19]. After NPPV treatment, AECOPD patients tended to generate uniform airway resistance against the breathing airflow and relatively continuous vibration energy. Therefore, their VEC and dynamic VRI image improved.

After NPPV treatment, a few healthy volunteers showed abnormities in the VRI image, such as desynchrony and unequal heights of EVP curves and an unsmooth MEF edge. This might be caused by desynchronization of the lung ventilation during NPPV. AECOPD patients may also suffer from desynchronization during NPPV; but in most cases, this may be hidden by the efficacy of NPPV therapy. The AVRIS increased rather than decreased in 4 patients after NPPV treatment and their clinical conditions did not improved, which might also be caused by the serious desynchronization. All the 4 patients had excessive secretions in the airway and serious pathological changes in the lungs. As a result, NPPV treatment is insufficient to improve the ventilatory function without a prior effectively anti-infective therapy or expectoration. This finding is consistent with our clinical experience that NPPV therapy, as recommended by guidebooks, should be avoided for patients with too much sputum.

The improved ventilatory function can be reflected by decline of the total AVRIS, which is significantly correlated with the decline of PCO_2_. However, it is noticeable that a decrease in the scores of sub-items does not necessarily suggest an improved ventilatory function but sometimes indicates a deteriorated ventilatory function. For instance, the inspiratory steep which appeared in the VEC of 11 AECOPD patients after NPPV treatment might only be related to the high-speed airflow of initial inspiration during positive pressure ventilation. In addition, another three AECOPD patients showed no dry or moist rales over lung auscultation and VRI examination before NPPV treatment. However, their dry and moist rales were found after NPPV treatment, which might be related to their amplified breath sound or to their improved ventilatory function. Therefore, in an AECOPD patient, attention should be paid not only to the changes in total or partial AVRIS but also to the clinical significance of each AVRIS component. As a result, the functional changes in ventilation reflected by the changes in AVRIS should be judged comprehensively. Further studies are needed to clarify this finding.

Several limitations of the present study should be acknowledged. Firstly, this is a single-center study with a relatively small sample size. Future studies with multicenter collaborations are warranted to verify our findings. Secondly, the VRI findings in this study correlated with clinical changes in HR and VAS (p < 0.05), as well as respiratory rate and AMUS (not significant). These observations did not support a clear-cut superiority of VRI over these indices. Nevertheless, our study showed that VRI can be at least a supplementary tool for earlier evaluation of the initial therapeutic effects in NPPV.

## Conclusions

In summary, VRI may quickly display the functional changes during NPPV treatment, and is highly correlated with the commonly-used clinical indices (such as PaCO_2_). In addition, VRI changes were in accordance with the physiological changes that reflected the ventilatory function during the NPPV treatment. Therefore, VRI is a promising noninvasive tool for evaluating the initial therapeutic effects of NPPV in AECOPD patients and predicting the success of NPPV in the early stage.

## Abbreviations

ITE: Initial therapeutic effect; NPPV: Noninvasive positive pressure ventilation; VRI: Vibration response imaging; AECOPD: Acute exacerbation of chronic obstructive pulmonary disease; AVRIS: Abnormal VRI scores; GOLD: Global initiative for chronic obstructive lung disease; VAS: Visual analogue scale; SAMU: Scale for accessory muscle use; VEC: Vibrational energy curve; MEF: Maximal energy frame; EVP: Expiratory vibration energy peak; QLD: Quantitative lung data; ICC: Intraclass correlation coefficient; LFE: Low and flat expiration; PEEP: Positive end-expiratory pressure.

## Competing interests

The authors declare that they have no competing interests.

## Authors’ contributions

Contributions of the authors include study design and supervising (Prof KJ), subject recruitment, VRI measurement, data collection, analysis and literature reviewing (DB, SL-f, TW, ZH-w). All authors have read and approved the final manuscript.

## Authors’ information

Dai Bing, PhD, Department of Respiratory Medicine, First Affiliated Hospital of China Medical University

Kang Jian, PhD, Department of Respiratory Medicine, First Affiliated Hospital of China Medical University

Sun Long-feng, MD, Department of Respiratory Medicine, First Affiliated Hospital of China Medical University

Tan Wei, BA, Department of Respiratory Medicine, First Affiliated Hospital of China Medical University

Zhao Hong-wen, PhD, Department of Respiratory Medicine, First Affiliated Hospital of China Medical University
